# The Newer Era of Veterinary Medicine1President’s Address delivered before the New York State Veterinary Medical Society, Brooklyn, N. Y., September 9, 1902.

**Published:** 1902-10

**Authors:** James Law

**Affiliations:** Ithaca, N. Y.


// THE JOURNAL

OF

COMPARATIVE MEDICINE AND VETERINARY ARCHIVES.

VOL. XXIII. *OCTOBER, 1902.* No. 10.

### THE NEWER ERA OF VETERINARY MEDICINE.[1]

BY PROF. JAMES LAW, F.R.C.V.S.,
ITHACA, N. Y.

IT is not always best to plume ourselves upon our achievements and the exalted position to which we have attained. It is liable to beget personal and professional pride, which is proverbially near to personal disaster. Yet, if we are duly impressed with the fact that we know so little, even to-day, and with the immeasurably vast field that still lies open to us for discovery and advancement, it is not unbecoming, and it may be a wholesome tonic and source of encouragement to take a cursory glance over our field of work, and note some of the changes that have taken place within the memory of living man.

But no one can confine such a glance to one field of achievement alone. The eye is at once caught and held by the wonders of modern progress on every side, which have kept pace with each other on every side of human enterprise.

The wonders of achievement of the past half century in every field of science have immeasurably surpassed those of any antecedent period of the world's history of many times its duration. If an accomplished man of the early part of the nineteenth century could return to mortal life furnished as he was at his exit, he would be made to feel at every step that the world was out of harmony with his habit of thought and experience. The triumphs of steam and electricity in bringing the most distant parts of the world to his door ; the revelations of the telescope and spectroscope in demon-

[1] President's Address delivered before the New York State Veterinary Medical Society, Brooklyn, N. Y., September 9, 1902.

strating the existence and composition of the greater part of the now visible stellar world, and in creating entirely new cosmic doctrines ; the regular succession of geologic strata and their relics of living forms furnishing a historic library of the earth's past history and a demonstration of the successive stages of its building. The demonstration of light and heat and electricity as forms of energy which may be made mutually interchangeable, and through which all the energies of the universe appear to be continually maintained ; the exhibition of the Röntgen light rays penetrating the most opaque organic matter and showing the existence, position, and form of the inorganic substance imbedded in its deepest recesses ; the responsiveness of given chemical compounds to light, through which we obtain the now commonplace but perfect photographic pictures of solid objects ; the power of charging given bodies with light or electric energy to be given off by slow degrees for useful ends ; the still more wonderful power of certain products —radius polonium—of radiating light indefinitely without the preliminary charging of them from a previously radiant source ; the transmission of electric messages, without solid conductor, through the ether for hundreds of miles, to be caught up and turned into words and thought by a receiver rightly attuned to their vibrations ; the submarine ship ; the dirigible flying machine. These are but a few of the more obvious advances that crowd in upon the average mind that is in no sense specialized in any one of these fields. To the scientist of 1800 these and the thousand other less obtrusive, but not less real, of the nineteenth century triumphs would come with a force that would be perfectly overwhelming.

When we turn to the advances in matters connected with medicine, the wealth of example is to us even more embarrassing. I can note but a very few of the salient points in which the present differs from the past.

When I took to the study of medicine the old days of vivisection had not quite passed ; every physician carried and used his lancet, and no veterinarian could get along without his fleam and blood stick. Every active inflammation, if not in its early stages, was held by many to be benefited by the free abstraction of blood. In Southern Europe the practice was even more firmly rooted ; under the then prevailing doctrines of Broussais, bleeding was advocated, "coup sur coup," time and again in the same attack, any increase of pulse, breathing, and mouth temperature being a warrant for a new abstraction. Broussais himself is reputed to have bled the babe at the breast and the old man of seventy. A number of years later the celebrated Italian statesman, Cavour, was held to be literally bled to a hopeless and fatal condition. By my time, however, the old and celebrated British physicians had become rather ashamed of their former excesses in this line, and the doctrine was already current that the type of disease had changed from a sthenic to an asthenic one, and that the former free depletions were not only not called for, but were in most cases absolutely injurious.

Why the type should have changed in Great Britain and not in France and Italy was not fully explained. Some of the incorrigibles and disloyal ones had a rankling suspicion that the violence of disease in former years was in no small degree due to the energy of the treatment applied. In any case the bent spring rebounded, and it flew so violently to the other side that the practitioner could hardly be made to confess that he knew of the existence much less the use of the instruments of vivisection.

To-day we are somewhat less prejudiced on either side. We recognize that no agency is more prompt and telling in its action than a diminution in the solids of the blood; for the water is soon supplied anew, and we have secured a transient abatement of suddenly induced plethora, a strong impression on the nervous system, and indirectly on circulation, nutrition, calorification, and the many recondite processes of cell-production, and destruction of metabolism, of hæmolysis, of leucocytosis, of diapedesis, of phagocytosis, and of chemotaxis, positive and negative.

If our theories are more complex, they are to-day based on a wider knowledge, and if we can no longer use the lancet as a panacea for all inflammatory, and many other diseases, we have intelligent views as to the small number of cases in which we can still draw on its use with striking advantage.

In the middle of the last century sedative treatment was the rule ; calomel and opium held a high place in the treatment of inflammatory affections, and the reliance upon it was so strong that it was naturally pushed to excess, and cases of mercurial salivation were frequently in evidence. To-day we still esteem calomel as a valuable antiseptic of the alimentary tract, favoring at the same time elimination from the whole chylopoietic system, to say nothing of its influence on metabolism and nutrition ; but we are no longer in danger of using it as a routine treatment, or of pushing its use to the extent of producing mercurialism.

A still more potent sedative was found in tartar emetic, which in combination with febrifuge diuretics was used as a routine treatment in febrile and inflammatory cases, for both man and beast. Here, again, we had an agent of great potency, especially in the carnivora and omnivora, which acted not only on the nervous system and indirectly on the skin and mucous membrane, quieting, relaxing, stimulating perspiration, but with an influence on the great liver and digestive functions. No doubt it oftentimes assisted wonderfully in abating the high nervous tension and inviting that resumption of secretions which was attended by a favorable crisis or lysis. But the blind reliance upon it as a routine treatment is gone, and when we now prescribe it it is only to secure some definite result of its physiological action, and that accomplished it is dropped from the prescription.

We might canvass other agents in the same way; but time presses, and I must let these stand as examples of a change at once radical and rational.

One great danger of to-day is that we should overlook the real worth of medicinal agents and methods that were standards of the great men of the past, and in so doing put from us much of the legacy they have left us. In my view of the matter wisdom lies in a certain respect for their methods which had, after all, a substantial basis, so that while entirely loyal to the advances of recent time, we should still stand ready to judicially pass on the medical lore of the past and make a right use of it in those cases in which it can be applied to advantage.

The ordinary diseases of bones in animals were formerly looked upon mainly as the result of mechanical injuries, and ringbones, and splints, and spavins were explained on the grounds of blows, concussions, sprains, and the like. To-day we look deeper, recognizing the bearing of heredity ; varying individual and family nutrition ; the quality and tone that come from appropriate diet and usage, and the lack of these with unsuitable food and disuse ; the influence of the poison that is found in the magnesian limestone, and of the impaired nutrition which comes in constitutional diseases. In short, to us an osteitis or periostitis is not simply a local inflammation to be subdued, but also, in many cases, a constitutional infirmity which must be corrected if we would make a permanent cure.

But in the whole field of medicine no advance has been so fundamental and fruitful as the introduction of microbian pathology. As a link with the past you have before you one who sat under the instruction of a man who even denied the existence of infection in epizootic diseases. William Dick, the founder of the Edinburgh Veterinary College, was in some respects a great man, according to his light. But in some lines his light was but gross darkness. Following Sydenham, he attributed all epizootic diseases to general causes—thermic, atmospheric, electrical, telluric—which assailed all animals alike, and to which those only succumbed which were at the time attuned to the morbid influence. For him rabies was the mere result of a general influence, and in his lectures he related how a rabid dog had licked a sore on his hand without producing the slightest evil result. For him lung plague was the result of inclement weather, exposure, poorly ventilated buildings, and the like, while rinderpest took its start from impaction of the third stomach, as a consequence of feeding on dry, fibrous, weathered and innutritious food. Dick stood long and loyally by his colors, logically applying his doctrines to all epizootic diseases ; he bore up bravely through the invasion of aphthous fever and lung plague, and it was only when the deadly rinderpest swept over the island that the old man, full of years and honors, had the chagrin to see his whole doctrine fall to pieces under the dread logic of facts.

The doctrine in question found in Dick at that time its most faithful exponent under all circumstances and as applied to all epizootics, but others clung tenaciously to general causes in the case of a number of communicable affections. Henri Bouley, of Alfort, a grand man, physically and mentally, a man who never touched a subject but to render it attractive, may be adduced as a striking example. Glanders was at that time recognized in England as contagious in all its forms and stages, even the indomitable Dick excluding it from his list of epizootic diseases due to general conditions. But in France contagion was accorded to acute glanders only, the chronic cases being held to be entirely free from infectious qualities. On both sides of the Channel the disease was attributed to general mismanagement and poor hygiene, as was tuberculosis at the same date. I have heard the silver-tongued Bouley discourse learnedly and to himself conclusively on the development of glanders from the impurities of stable air, insufficient or injurious food, cold and damp, and, above all, from overwork, the creatin, creatinin, and other morbid products retained in excess within the system, laying the foundation of the deadly morbid processes. The results of the two doctrines were not a little remarkable.

In Great Britain it was rather rare to see a glandered horse, while in the veterinary school at Alfort they were daily on exhibition. In the English army glanders was virtually unknown, and the occurrence of a single case was held to reflect seriously on the regimental veterinarian, while in the French army 9 per cent. per annum became victims of this disease.

But even when contagion was recognized the mystery continued as to the actual agent by which the transmission took place. This ignorance was cloaked under such vague and speculative terms as "miasen," "humor," "virus," and, later, "morbid functions in cells," and pathogenic "bioplasen."

It was only when Pasteur in his researches on alcoholic fermentations demonstrated the facts that such fermentations, and even putrefactions, were the direct consequence of the multiplication in the liquids of particular living organisms or ferments, that the foundation had been laid for a true doctrine of contagion.

Lister, at that time assistant surgeon to the Edinburgh Surgical Hospital (and whose lectures I then availed myself of), conceived the idea that the fermentation and putrefaction changes in wounds, and notably the terrible hospital gangrene, must be due to similar ferments, and, therefore, preventable. He pursued his research for seven years, and in 1877 was enabled to report the demonstration of the truth of his convictions. It was years, however, before it made headway in other lands, Guerin, for example, taking the lead in Paris in 1871; but very soon the scourge of the hospital came to be held as a thing of the past, and patients went to the operating-table with a hopefulness and confidence that had never before been known.

Prior to Lister's researches compound fractures and gunshot wounds were most dangerous contingencies, and most operations involving the opening of the abdomen, the cranium, or the chest were considered very redoubtable; whereas to-day they are undertaken light-heartedly and in a most hopeful spirit. And yet long before Lister's day the castration of females had been generally practised in different countries, often by others than veterinarians (by gelders and shepherds), with almost invariable success, and the trephining of the cranium for cœnurus was a common and often a successful practice with veterinarians. The danger lay more in the concentration of the infection in great hospitals than in the dangerous nature of the lesion by itself. The virtual disappearance of hospital gangrene and other wound infections from city hospitals is one of the most splendid monuments that could be raised to the memory of a man. No matter if we have largely discarded Lister's antiseptic surgery, substituting wherever possible aseptic treatment, the same principle underlies both, and it is to Lister that we must attribute the planting of the germ that has borne such a splendid fruition.

As Lister first applied the great discovery of Pasteur to surgery, so Davaine may be said to have done for medicine, and it is an important fact that it was in the line of medicine of animals. Pollender in 1849 had found multitudes of very fine rods in the blood of anthrax-stricken cattle. Davaine confirmed this a year later, and Brauel seven years later still. But it was only in 1863, and as a result of the Pasteur discovery, that it suddenly occurred to Davaine that this was the true cause of the disease. But anthrax was still a very indefinite quantity. By a reference to the literature of the time it will be seen that it included black quarter, malignant œdema, hog cholera, swine plague, swine erysipelas, the various hemorrhagic septicæmias, and even Texas fever. It was only slowly and by patient work in the field and laboratory that these various independent affections were differentiated and each assigned its place according to its true microbian cause and pathology. The final demonstration has come in every case through the discovery of the microbe, and gradually veterinary pathology has been enriched by the addition of a long list of animal diseases, all having much in common and all due to different microbian causes. The laboratory study of these affections has not only given us the demonstrations of this large class of maladies, but it has contributed largely to the identifying of many deadly forms of poisoning by toxins, ptomaïnes, and enzymes, and again by mineral agents like powdered soaps added to swill, and which have often proved as deadly to the herds poisoned in this way as the most destructive forms of hemorrhagic septicæmias or genuine swine fevers. The localization of such outbreaks, their confinement strictly to certain filthy enclosures, to herds fed in a particular way, and the absence of the specific germ of the disease which is suspected to exist, will usually furnish the crucial test, and differentiate for scientific and for practical sanitary purposes, affections which, in time past, would have been classed together as one common malady. So far as these maladies are concerned, we can to-day place veterinary sanitary science on a sound basis, always provided that its administrators are selected for their knowledge of modern animal pathology and are not mere office-holders appointed in recognition of personal claims of other kinds. Closely connected with these affections are rinderpest, sheep-pox, and lung plague, which, since the extinction of the latter in this republic, we have come to think of as plagues of the old world which can never more directly interest the favored stock-owners of America. But our acquisition of the Philippines has brought us into direct contact with Asia, the ancestral home of animal plagues, and the war in those islands has apparently been the immediate cause of the wide and deadly extension of the plague of the steppes into the herds of the Filipinos. Fortunately for our home herds, our vigilant Bureau of Animal Industry was quick to see and meet the danger in securing an order of exclusion of all cattle and pigs coming from the Philippines, from the United States, and any of its dependencies.

But as veterinarians of the imperial country we can no longer afford to ignore the rinderpest of the old world. It is now domiciled on land which in the progress of events has come to be a part of our nation, and we are called upon in right and justice to assist the islanders in ridding themselves of a scourge of which our interference was, in a large measure, the occasion. Manila must look to America for the means of extinction of this plague which now threatens the ruin of her cattle industry, and the American veterinarian must stand ready to take up the work intelligently and to carry it through to a successful issue. No cattle plague is more redoubtable and deadly, and yet for those who may be charged with its extinction the prospect is much more hopeful than when we are dealing with a pestilence which is slower in its progress, more liable to prove occult in its course, and in dealing with which every step is liable to be clogged and even blocked by a badly informed and often prejudiced legislative body. In a dependency like the Philippines, under an intelligent centralized authority, there is a better prospect of the adoption of effective measures of extinction and of carrying these through without varying or hesitation to a splendid success. If this can be accomplished, and if the object lesson can be taken in the right spirit by the United States, encouraging them to grapple effectively with their own indigenous plagues, the blood and treasure expended on those distant islands may be repaid many times over in the future prosperity of our animal industry.

(To be continued.)

---

State Veterinarian Pearson, of Pennsylvania, after visiting parts of Iowa and Wisconsin, returned to his post about the middle of September.

